# Termination Rules for Variable-Length CD-CAT From the Information Theory Perspective

**DOI:** 10.3389/fpsyg.2019.01122

**Published:** 2019-05-29

**Authors:** Lei Guo, Chanjin Zheng

**Affiliations:** ^1^Faculty of Psychology, Southwest University, Chongqing, China; ^2^Southwest University Branch, Collaborative Innovation Center of Assessment toward Basic Education Quality, Chongqing, China; ^3^Chongqing Collaborative Innovation Center for Brain Science, Chongqing, China; ^4^Department of Educational Psychology, Faculty of Education, East China Normal University, Shanghai, China; ^5^Words Up Your Way, Beijing, China

**Keywords:** computerized adaptive testing, cognitive diagnostic model, information theory, Shannon entropy, Kullback–Leibler distance, variable-length CD-CAT

## Abstract

Cognitive diagnostic computerized adaptive testing (CD-CAT) aims to take full advantage of both cognitive diagnosis (CD) and CAT. Cognitive diagnostic models (CDMs) attempt to classify students into several attribute profiles so as to evaluate their strengths and weaknesses while the CAT system selects items from the item pool to realize that goal as efficiently as possible. Most of the current research focuses on developing the item selection strategies and uses a fixed-length termination rule in CAT. Nevertheless, a variable-length termination rule is more appropriate than the fixed-length rule in order to bring out the full potential of CD-CAT. The current study discussed the inherent issue of instability over different numbers of attributes with the previous termination rules (the Tatsuoka rule and the two-criterion rule), proposed three termination rules from the information theory perspective, and revealed the connection between the previous methods and one of the information-based termination rules that will be discussed, further demonstrating the instability issue. Two simulation studies were implemented to evaluate the performance of these methods. Simulation results indicated that the SHE rule demonstrated strong stability across different numbers of attributes and different CDMs and should be recommended for application.

## Introduction

The goal of cognitive diagnosis is to obtain the students' status of mastering specific attributes measured by items in psychological and educational assessment. In recent decades, various cognitive diagnosis models (CDMs) have been developed to evaluate the attribute profiles or latent classes for each student, which designates whether each of the measured attributes or skills has been mastered (Tatsuoka, 1983; Mislevy et al., 2000; Junker and Sijtsma, 2001; Rupp et al., 2010).

One main application of CDM that has been published by many researches is in combination with computerized adaptive testing (CAT), which can be termed as cognitive diagnostic computerized adaptive testing (CD-CAT; Cheng, 2009; Huebner, 2010). The major benefit of CAT is that a tailored test can be generated for each individual *via* selecting items from the item pool according to their responses to previous items. Generally speaking, CAT will get the same precision of ability estimation as a traditional paper and pencil test by using fewer items. In other words, CAT can provide a high-efficient estimate for latent trait of interest (Weiss and Kingsbury, 1984). Thus, it is obvious that CD-CAT may have a performance comparable to Item Response Theory (IRT)-CAT.

To date, numerous studies have been done to examine the property of CD-CAT (Cheng, 2009; Wang et al., 2011, 2012; Wang, 2013; Kaplan and de la Torre, 2015; Zheng and Wang, 2017). However, most previous studies focused on proposing item selection strategies and almost used the fixed-length rule to stop the CD-CAT. It is possible to implement a needlessly long test to some students and an undesirably short test to others when the fixed-length termination rule is adopted. Consequently, it often leads to different measurement precision for different students. In practice, one may prefer that every student has nearly the same degree of estimate precision, which is a major strength of CAT over non-adaptive testing (Weiss and Kingsbury, 1984). The termination rule issue in CD-CAT has begun to attract some attention from researchers. Tatsuoka (2002) suggested that the CD-CAT stops when the examinee's posterior probability of a given attribute profile exceeded 0.80 (hereafter denoted as the Tatsuoka rule). Hsu et al. (2013) proposed a two-criterion termination rule by adding another criterion to the Tatsuoka rule. Cheng (2008) mentioned the possibility of proposing termination rules from the information theory perspective, but no theoretical explanation or empirical study was provided. The current study demonstrates the derivation of three termination rules from the information theory perspective and evaluates the termination rules using simulation studies.

In the following, first, the previous methods (i.e., the Tatsuoka rule and the two-criterion rule) for variable-length CD-CAT are summarized and their inherent issue of instability over different numbers of attributes will be discussed. Second, we introduce three information-based termination rules for CD-CAT. The connection between the previous methods and one of the information-based termination rules is shown, which further demonstrates the instability issue. Third, following this, two simulation studies are conducted to assess the performance of the new termination rules over different numbers of attributes and CDMs with regard to the instability issue. Finally, some important issues in variable-length termination rules will be discussed.

## The Previous Rules for Variable-Length CD-CAT and Their Issues

To our knowledge, two termination rules for CD-CAT have been proposed, namely, the Tatsuoka rule and the two-criterion rule, respectively. Tatsuoka (2002) suggested that a CD-CAT stops when the examinee's posterior probability of a given attribute profile exceeded 0.80, i.e., the posterior probability of one latent class (PPLS) is bigger than 0.80. The principle is that the more peaked the posterior probability distribution is, the more dependable the classification is (Huebner, 2010). Inspired by the Tatsuoka rule, Hsu et al. (2013) recommended to add another criterion for the second largest PPLS. Thus, the modified termination rule for variable-length CD-CAT using the following two criteria were proposed:

Criterion 1: CD-CAT will be stopped when the largest PPLS is not smaller than a predetermined value (e.g., 0.70).

Criterion 2: CD-CAT will be stopped when the largest PPLS is not smaller than a predetermined value (e.g., 0.70) and the second largest PPLS is not larger than a predetermined value (e.g., 0.10).

The key of the two-criterion rule is to determine the threshold for the second largest PPSL. The following formula can be used to determine the lower bound and upper bound for the second largest PPSL.

(1) $$\begin{array}{l} P_{2nd}=\frac{1-P_{1st}}{2^{K}-1}+\frac{\left(2^{K}-2\right)\times \left(1-P_{1st}\right)\times d}{2^{K}-1},0\leq d\leq 1 \end{array}$$

where *P*_1st_ and *P*_2nd_ are the prespecified largest and second largest PPSL, *K* represents the number of attributes, and *d* is the weighted value for *P*_2nd_. Based on the simulation results, Hsu et al. (2013) offered two suggestions:

1. One can set the value of *P*_1st_ as high as 0.90 or 0.95 if the high-stakes tests are implemented. Thus, only Criterion 1 will be needed and Criterion 2 is not necessary.
2. One can set the value of *P*_1st_ at 0.70 or lower, and the *d* value can be set between 0.25 and 0.50, or simply set *P*_2nd_ = 0.10 if the low-stakes tests are implemented.

The Tatsuoka rule is intuitive and simple, but with an increase in the number of attributes, which leads to the exponential increase of the number of attribute profile, it discards more and more information contained in the other attribute profiles since it only cares about the one with the largest probability mass. It is a sensible conjecture that there is an unstable issue with the Tatsuoka rule, namely, the realized accuracy of the attribute profile estimate might not be consistent across different numbers of attributes under the same model by implementing the Tatsuoka rule. Hsu et al. (2013) recognized this fact and attempted to solve this issue by setting a lower and upper bound for the second largest PPSL. One of the factors that influence the determination of the second largest PPSL is the number of attributes. But the fine-tuning of the second largest PPSL is of *ad hoc* nature, which makes the implementation difficult. The practical recommendation for *d* or *P*_2nd_ taking a value of 0.1 can ameliorate this problem, but it was made based on the simulation study for only the case of six attributes and it may bring the instability issue again.

The current study proposes some new termination rules from the information perspective and evaluates their performance for different numbers of attributes under two major CDMs. Statistically speaking, the development of termination rules for CD-CAT aims to identify some statistical tools that describe certain characteristics of the posterior distribution of cognitive profiles. Both Tatsuoka and Hsu and his associates used the point(s) in the distribution with the largest concentration of the probability mass and discarded the remaining, and thus their methods can be labeled as a partial information approach. It is also worth pointing out that Tatsuoka did not carry out any empirical simulation study on the termination rule he proposed and Hsu et al. (2013) did not explore the performance of the two-criterion rule for different numbers of attributes, although it is an important factor in Equation (1). Another more powerful tool that describes a distribution is information indexes, which can capture the characteristics of the whole distribution and thus can be considered as a full information approach. The major advantage of the new methods is that they incorporate the information of several attributes easily and they are expected to provide a simple consistent termination rule without demanding delicate fine-tuning as the two-criterion rule requires.

## Information Theory for CDM and Information-Based Termination Rules for CD-CAT

### Information Theory for CDM

A brief introduction to information theory, which is heavily borrowed from Cover and Thomas (2012) and Chang et al. (2016), is given below. Since the models involved in cognitive diagnosis are discrete, only the discrete version of various information indexes is presented where possible.

Information was first introduced by Fisher (1925). An important development in the information theory, introduced by Shannon (1948), was that of entropy. Shannon entropy is used to describe the uncertainty in the distribution of a random variable. Specifically, its value becomes maximum when distribution is uniform and minimum when distribution is a single point mass. In cognitive diagnosis, we need to classify an examinee into a certain attribute profile, so the posterior distributions were expected to be a point mass. This means the smaller the Shannon entropy value is, the more accurate the classification is. Let $\alpha_{c}=\left(\alpha_{c1},\alpha_{c2},\ldots ,\alpha_{cK}\right)\;\left(c=1,\ldots ,2^{K}\right)$ be the attribute profile of examinees, and there were 2^*K*^ attribute profiles totally. Let $\pi =\left(\pi_{1},\pi_{2},\ldots ,\pi_{2^{K}}\right)$ represent the posterior probability vector, and the element π_*c*_ is corresponding probability for **α**_*c*_. Note that $\pi_{c}>0\;\left(c=1,\ldots ,2^{K}\right)$, and $\sum_{c=1}^{2^{K}}\pi_{c}=1$. The Shannon entropy of **π** is expressed as follows:

(2) $$\begin{array}{l} H\left(\pi \right)=\sum_{c=1}^{2^{K}}\pi_{c}\log\left(1/\pi_{c}\right) \end{array}$$

The notion of entropy was extended to relative entropy by Kullback and Leibler (1951) and thus it was also denoted as the Kullback–Leibler (KL) distance. The relative entropy *KL*(*p*||*q*) measures the divergence between distributions *p* and *q*. Cover and Thomas (2012) gave the original expression for the KL distance, i.e., $KL\left(p||q\right)=E_{p}\left[\log\frac{p\left(x\right)}{q\left(x\right)}\right]$. KL distance is non-negative and equals zero if distributions *p* and *q* are identical and becomes large as the distributions diverge. In cognitive diagnosis, the KL distance between *Y*_*ij*_ conditioning on estimated attribute profile $f(Y_{ij}|\overset{⌢}{\alpha })$ and the conditional distribution of *Y*_*ij*_ given another attribute profile **α**_*c*_, i.e., *f*(*Y*_*ij*_|**α**_*c*_), is expressed as follows:

(3) $$KL_{j}(\overset{⌢}{\alpha }||\alpha_{c})=\sum_{y=0}^{1}\log\left(\frac{P(Y_{ij}=y|\overset{⌢}{\alpha })}{P(Y_{ij}=y|\alpha_{c})}\right)P(Y_{ij}=y|\overset{⌢}{\alpha })$$

where *Y*_*ij*_ is the response of 1 (correct) and 0 (incorrect) to item *j* for examinee *i*. The larger the KL index value is, the more accurate the classification is.

The distinct difference between Shannon entropy and KL distance is that Shannon entropy uses some absolute values to describe one distribution while the KL distance tries to capture the distance between two distributions; thus, we can develop information-based terminations rules from this absolute-vs.-relative perspective.

## Information-Based Termination Rules for CD-CAT

Some work has been done to develop item selection algorithms for CD-CAT from the information perspective (Xu et al., 2003; Cheng, 2009). The derivation of the termination rules from the information perspective is straightforward and can be obtained by simply replacing the random variable by the posterior distribution of the attribute profiles. The information-based termination rules suggest that a test can stop when:

- The Shannon entropy of the posterior distribution becomes reasonably small (denoted as the SHE rule):
  (4) $$\begin{array}{l} H\left(g_{t}\right)<\varepsilon \end{array}$$
  where *g*_*t*_ is the corresponding posterior distribution when an examinee answers *t* items. ε ∈ *R*^+^ is a very small positive number. The SHE rule is equivalent to verify that the uncertainty of the posterior distribution has been reduced to a prescribed absolute level (obviously, this falls into the category of the absolute approach). Most of the posterior mass density is more concentrated and a few points (attribute profiles in CDM) occupy majority of the probability in posterior distribution. Because we hope one attribute profile will take up most of the probability, the CD-CAT test stops when Equation (4) is satisfied.
- The KL distance (relative entropy) between two adjacent posterior distributions becomes small enough (denoted as the KL-distance rule):
  (5) $$\begin{array}{l} D\left(g_{t}||g_{t-1}\right)<\varepsilon \end{array}$$
  where *g*_*t*−1_ is the posterior distribution of attribute profiles after (*t*−1) items have been administered. The rationale for the KL-distance rule is that if the posterior distribution change between responding *t* items and (*t*−1) items is negligible, the final attribute profile will be confirmed. Thus, the CD-CAT test stops when Equation (5) is satisfied.
- The change of Shannon entropy for the adjacent posterior distributions becomes reasonably small (denoted as the SHE-difference rule):
  (6) $$\begin{array}{l} |H\left(g_{t}\right)-H\left(g_{t-1}\right)|<\varepsilon \end{array}$$
  The SHE-difference rule and the KL-distance rule follow a similar line of thinking and both of them fall into the category of the relative approach. Both of them involve the comparison of the two adjacent posterior distributions with the test stopping when the difference between the posterior estimate and the immediate previous one is small enough to reach a predetermined level; i.e., the posterior estimate for the true attribute profile cannot be significantly improved given the current estimate and item selection method.

In summary, the above three information-based rules introduced in this section can fall into two categories: an absolute approach and a relative approach. The SHE rule is an absolute approach while the other two are relative approaches.

## The Connection and Difference Between the Previous Rules and the SHE Rule

The Tatsuoka rule and the two-criterion rule can be re-expressed as the SHE rule. This reformulation can further demonstrate the issues with the previous methods discussed above. For the Tatsuoka rule, *P*_1st_ is required to be larger than 0.8. This is equivalent to the following:

1. The addend for *P*_1st_ in the SHE rule is required to be smaller than
  (7) $$\begin{array}{l} 0.8*\log_{e}\left(\frac{1}{0.8}\right)=0.179 \end{array}$$
2. The remaining probabilities excluding *P*_1st_ satisfies the assumption that

(8) $$\begin{array}{l} \overset{2^{K}-1}{\underset{i=1}{\sum }}P_{i}=1-0.8=0.2 \end{array}$$

In other words, if the preset value for *P*_1st_ has been set at 0.8, the remaining 2^*K*^−1 attribute profiles share the rest of probability. In the worst case, the 2^*K*^−1 attribute profiles share 0.2 equally, which signifies that they are equally probable. Thus, the Shannon entropy value of this probability distribution equals $0.8*\log_{e}\left(\frac{1}{0.8}\right)+\left(\frac{0.2}{2^{K}-1}*\log_{e}\left(\frac{2^{K}-1}{0.2}\right)\right)*\left(2^{K}-1\right)$. In the best scenario, the second largest PPSL (*P*_2nd_) takes all the probability mass 0.2, i.e., the remaining probabilities are all 0s; the Shannon entropy value of this probability distribution equals $0.8*\log_{e}\left(\frac{1}{0.8}\right)+0.2*\log_{e}\left(\frac{1}{0.2}\right)=\text{0}\text{.5}$.

Two important observations can be made. First, a certain termination criterion value of the Tatsuoka rule corresponds to an interval of the SHE rule, and the range only depends on the number of attributes. Table 1 shows the various ranges and the lower (upper) bound in the SHE rule under different numbers of attributes when *P*_1st_ is set at 0.7 or 0.8 in the Tatsuoka rule.

**Table 1:** Correspondence between the Tatsuoka and the SHE rule under different numbers of attributes.

***P*_**1st**_**	**Number of attributes**	**Shannon entropy**		
		**Lower bound**	**Upper bound**	**Range**
0.7	4	0.611	1.423	0.812
	5	0.611	1.641	1.030
	6	0.611	1.854	1.243
	7	0.611	2.064	1.453
	8	0.611	2.273	1.662
0.8	4	0.500	1.042	0.542
	5	0.500	1.187	0.687
	6	0.500	1.329	0.829
	7	0.500	1.469	0.969
	8	0.500	1.609	1.109

As shown in Table 1, the lower bound is always a constant when *P*_1st_ is set at a fixed value. However, the upper bound and the range of Shannon entropy rely on the number of attributes. Specifically, with the increase of the attribute number, the upper bound and range become larger. Consequently, for one particular Tatsuoka rule criterion, the larger the range is, the more possible values the classification accuracy can take.

Second, there is considerable overlap for the interval for two neighboring Tatsuoka rule values. For example, the lower and upper bounds are 0.611 and 2.273, respectively, when *P*_1st_ = 0.7 (*K* = 8), and the values become 0.5 and 1.609 when *P*_1st_ = 0.8 (*K* = 8). The size of overlaps is 0.998 (= 1.609–0.611). The overlap implies that the finalized classification accuracy might be similar or reversed (a higher classification accuracy rate for a lower criterion) for two different Tatsuoka rule criteria, which is undesirable.

It is clear that the Tatsuoka rule is not as refined as the SHE rule. The final realized classification accuracy of one particular criterion from the Tatsuoka rule may vary depending on how many attributes there are. This correspondence between the two methods further reveals the root cause of the instability issue with the Tatsuoka rule.

A similar reformulation can be done for the two-criterion rule. The two-criterion rule with *P*_1st_ = 0.7 and *P*_2nd_ = 0.1, in terms of the SHE rule, is equivalent to the following:

1. The addend for the *P*_1st_ in the SHE rule is smaller than
  (9) $$\begin{array}{l} 0.7*\log_{e}\left(\frac{1}{0.7}\right)=0.250 \end{array}$$
2. The addend for the *P*_2nd_ and other addends in the SHE rule are smaller than
  (10) $$\begin{array}{l} 0.1*\log_{e}\left(\frac{1}{0.1}\right)=0.230 \end{array}$$
3. The remaining probabilities excluding *P*_1st_ and *P*_2nd_ satisfy the assumption that.
  (11) $$\begin{array}{l} \overset{2^{K}-2}{\underset{i=1}{\sum }}P_{i}=1-0.7-0.1=0.2 \end{array}$$

Following the same line of reasoning, correspondence between the two-criterion rule and the SHE rule under different numbers of attributes can also be derived. Table 2 shows the various ranges and the lower (upper) bounds in the SHE rule under different numbers of attributes when *P*_1st_ is set at 0.7 or 0.8 and *P*_2nd_ is fixed at 0.1 in the two-criterion rule.

**Table 2:** Correspondence between the two-criterion rule and the SHE rule under different numbers of attributes.

***P*_**1st**_**	***P*_**2nd**_**	**Number of attributes**	**Shannon entropy**		
			**Lower bound**	**Upper bound**	**Range**
0.7	0.1	4	0.802	1.343	0.541
		5	0.802	1.489	0.687
		6	0.802	1.630	0.828
		7	0.802	1.771	0.969
		8	0.802	1.910	1.108
0.8	0.1	4	0.639	0.910	0.271
		5	0.639	0.982	0.343
		6	0.639	1.053	0.414
		7	0.639	1.123	0.484
		8	0.639	1.193	0.554

Similar observations can be made, although there is some reduction in the size of the corresponding SHE interval for the two-criterion rule and their overlap.

In summary, all the termination rules can be summarized in a new taxonomical framework as in Table 3. It provides a basis for better understanding and discussion of the advantages and disadvantages of all methods, and the following two simulation studies will be designed to evaluate the absolute-vs.-relative and partial-vs.-full information comparison, respectively.

**Table 3:** The taxonomy for the termination rules.

	**Partial information**	**Full information**
Absolute approach	Tatsuoka rule Two-criterion rule	SHE rule
Relative approach	–	SHE-difference rule KL-distance rule

## Simulation Studies

### The DINA and Fusion Model

Two commonly used CDMs are the fusion model (Hartz, 2002) and the Deterministic Input; Noisy And gate (DINA) model (Junker and Sijtsma, 2001). An essential component underlying CDMs is the **Q**-matrix (Tatsuoka, 1983). Assume a test contains *J* items and *K* attributes, the **Q**-matrix is usually defined as a *J* × *K* matrix. The element that is related to the *k*th attribute for the *j*th item can be written as *q*_*jk*_. *q*_*jk*_ = 1 if item *j* measures the attribute *k*, and *q*_*jk*_ = 0 otherwise.

The DINA model assumes that only when the examinee has mastered all attributes required by the item can he respond correctly. In fact, two possible behaviors, namely, “slip” and “guess,” may occur when examinees respond to the items. Slip represents that the examinee gives an incorrect response to the item even though (s)he has mastered all the required attributes of this item, and guess indicates that the examinee gives a correct response to the item even though (s)he has not mastered all the required attributes of this item. With these characteristics, the correct response probability to the *j*th item for the *i*th examinee is

(12) $$\begin{array}{l} P\left(Y_{ij}=1|\alpha_{\text{i}}\right)={\left(1-s_{j}\right)}^{\eta_{ij}}{g_{j}{}^{1-}}^{\eta_{ij}} \end{array}$$

where **α**_**i**_ = (α_*i*1_, α_*i*2_, …, α_*iK*_) is the attribute profile of examinee *i*. α_*ik*_ = 1 if *i*th examinee possesses attribute *k*, and α_*ik*_ = 0 otherwise. *s*_*j*_ and *g*_*j*_ are the slip parameter and guess parameter, respectively. $\eta_{ij}=\prod_{k=1}^{K}{\alpha_{ik}}^{q_{jk}}$ is a latent variable that represents the examinee *i*'s ideal response to item *j*. Note that if examinee *i* has mastered all the required attributes of item *j*, η_*ij*_ = 1; otherwise, η_*ij*_ = 0.

To introduce the fusion model, two types of item parameters are needed to be defined first: a) the parameter $\pi_{j}^{*}$ denotes the probability of correct response to item *j* if examinees have mastered all measured attributes, and b) the parameter $r_{jk}^{*}$ denotes the penalty for not having mastered attribute *k* of item *j*. Thus, the correct response probability in the fusion model arrives as

(13) $$\begin{array}{l} P\left(Y_{ij}=1|\alpha_{\text{i}},{\pi_{j}}^{*},{r_{jk}}^{*},c_{j}\right)={\pi_{j}}^{*}\overset{K}{\underset{k=1}{\prod }}{r_{jk}}^{*\left(1-\alpha_{ik}\right)q_{jk}}P_{c_{j}}\left(\theta_{i}\right) \end{array}$$

where *P*_*c*_*j*__(θ_*i*_) is the Rasch model in which the item difficulty parameter is *c*_*j*_ and θ_*i*_ is the ability parameter for examinee *i* to explain the additional contribution except those specified attributes in the ***Q***-matrix. Usually, *P*_*c*_*j*__(θ_*i*_) is set at 1 (Henson and Douglas, 2005; McGlohen and Chang, 2008; Wang et al., 2011). With this constraint, the fusion model becomes the Reduced Reparameterized Unified Model [R-RUM; (Hartz, 2002)]. This practice is adopted in this study.

### Item Selection Method

Xu et al. (2003) introduce the KL distance into CD-CAT and use the KL index as an item selection strategy. In order for KL distance to be able to indicate item *j*'s global discrimination power between $f(Y_{ij}|\overset{⌢}{\alpha })$ and all possible attribute profiles, the KL index was proposed to describe the sum of KL distance between $f(Y_{ij}|\overset{⌢}{\alpha })$ and all *f*(*Y*_*ij*_|**α**_*c*_)s:

(14) $$KL_{j}(\overset{⌢}{\alpha })=\sum_{c=1}^{2^{K}}\left[\sum_{y=0}^{1}\log\left(\frac{P(Y_{ij}=y|\overset{⌢}{\alpha })}{P(Y_{ij}=y|\alpha_{c})}\right)P(Y_{ij}=y|\overset{⌢}{\alpha })\right]$$

where *K* is the number of attributes, and there will be 2^*K*^ possible attribute profiles.

The item with the maximum KL value, given the attribute profile of $\overset{⌢}{\alpha }$, will be administered from the item pool. Furthermore, to feature the different importance of different attribute profiles, the supplement in Equation (14) is weighted by the posterior probability, and this modification can be called PWKL information (Cheng, 2009). The selection criterion in PWKL information is expressed as follows:

(15) $$PWKL_{j}(\overset{⌢}{\alpha })=\sum_{c=1}^{2^{K}}\left\{\sum_{y=0}^{1}\left[\log\left(\frac{P(Y_{ij}=y|\overset{⌢}{\alpha })}{P(Y_{ij}=y|\alpha_{c})}\right)P(Y_{ij}=y|\overset{⌢}{\alpha })\right]g(\alpha_{c}|y_{t-1})\right\}$$

where $g(\alpha_{c}|y_{t-1})=p(\alpha_{c}){\prod^{\;}}^{\;}{}_{j=1}^{t-1}P{\left(Y_{ij}=1|\alpha_{c}\right)}^{yij}[1-P{\left(Y_{ij}=1|\alpha_{c}\right)}^{1-yij}$ is the posterior probability of **α**_*c*_, *p*(**α**_*c*_) is the prior probability, and *y*_*t*−1_ is the response vector for examinee *i* on previous *t* – 1 items.

### Study 1: Absolute vs. Relative Approach

#### Design

The item pool consisted of 300 items and no maximum test length was imposed in order to investigate the performance of all methods without any constraints. Each attribute was set to be measured by 40% of all items and make sure that each item at least measured one attribute. For the DINA model, the *s*_*j*_ and *g*_*j*_ parameters were both generated from *U*(0.05, 0.25). For the fusion model, the item parameters ${\pi^{*}}_{j}$ and ${r_{jk}}^{*}$ were generated from *U*(0.75, 0.95) and *U*(0.2, 0.95), respectively (Henson and Douglas, 2005). A total of 2,000 examinees were generated assuming that every examinee has 50% probability of mastering each attribute. That is, there were 64 equally distributed attribute profiles in the population if a test measured six attributes.

The major goal of this simulation was to evaluate the stability of absolute and relative approaches across different numbers of attributes and different CDMs. Three factors were manipulated in this study. First, there were two models used in the study: the DINA model and the fusion model. Second, the number of attributes varied from 4 to 8. Finally, three information-based termination rules were investigated. The Tatsuoka rule and the two-criterion rule as partial information absolute methods were also included as baselines. For the SHE-difference rule and KL-distance rule, there were five levels for ε : 0.01, 0.05, 0.1, 0.15, and 0.2. Levels for ε were set at 0.3, 0.6, 0.9, 1.2, 1.5, and 1.8 for the SHE rule. The termination criterion for the Tatsuoka rule *P*_1st_ was set as either 0.5, 0.6, 0.7, 0.8, or 0.9, while for the two-criterion method, the criterion for *P*_2nd_ to be set as 0.1 was added as well. Thus, there were (5 + 5 + 5 + 5 + 6) × 5 × 2 = 260 conditions.

The major dependent variables were the same as in Hsu et al. (2013). Say: (a) classification accuracy of attribute profiles, pattern correct classification rate (PCCR), calculated as the percentage of examinees whose attribute profiles were estimated correctly. For the interpretation of the result, we care more about the stability of PCCR for one particular termination criterion for different numbers of attributes and CDMS than PCCR itself; and (b) the test length at the end of the CD-CAT.

#### Results

Tables 4–**8** show the PCCR values and the concerned statistics, such as mean (M), standard deviation (SD), maximum (Max), and minimum (Min) of the test length at the end of the CD-CAT across all examinees. The results are summarized as the following.

**Table 4:** Classification accuracy for attribute profile and test length using the Tatsuoka rule.

**#Attribute**	**P1st**	**DINA**					**FM**				
		**M**	**SD**	**Max**	**Min**	**PCCR**	**M**	**SD**	**Max**	**Min**	**PCCR**
4	0.9	7.1	2.1	20	3	0.941	12.7	5.2	42	6	0.926
	0.8	5.5	1.6	18	2	0.869	9.9	4.2	33	5	0.869
	0.7	4.5	1.1	14	2	0.780	7.3	2.7	23	4	0.779
	0.6	4.3	0.9	11	2	0.739	6.4	2.4	31	4	0.725
	0.5	4.1	0.7	5	2	0.752	5.7	2.0	15	3	0.665
5	0.9	9.3	2.8	27	4	0.934	17.6	7.3	95	7	0.922
	0.8	7.6	2.2	21	3	0.863	13.9	5.2	41	5	0.853
	0.7	6.8	2.0	17	3	0.799	11.1	4.1	41	5	0.788
	0.6	5.5	1.1	14	3	0.737	9.4	3.6	28	4	0.714
	0.5	5.2	1.0	14	3	0.698	7.5	2.6	28	3	0.664
6	0.9	12.9	12.3	300	5	0.935	23.1	15.3	300	9	0.923
	0.8	10.6	10.3	300	4	0.856	18.0	7.5	70	7	0.855
	0.7	8.7	2.7	24	4	0.773	15.5	6.5	59	6	0.777
	0.6	7.2	1.8	25	4	0.732	11.4	4.2	38	5	0.705
	0.5	6.4	1.0	15	4	0.679	11.4	4.5	37	4	0.657
7	0.9	19.4	15.0	300	6	0.925	31.9	25.0	300	9	0.931
	0.8	14.2	12.8	300	6	0.842	24.5	11.8	117	8	0.853
	0.7	9.9	2.2	21	6	0.757	18.4	5.4	57	10	0.765
	0.6	10.6	3.9	44	5	0.723	16.9	6.9	73	6	0.703
	0.5	8.0	1.7	21	4	0.672	14.6	6.0	56	6	0.649
8	0.9	33.8	17.3	300	6	0.926	45.3	40.3	300	10	0.926
	0.8	24.1	14.9	300	6	0.845	33.3	25.1	300	9	0.847
	0.7	13.9	4.9	43	5	0.767	25.7	13.7	131	7	0.764
	0.6	11.5	3.8	36	5	0.708	22.2	10.6	115	7	0.712
	0.5	11.3	3.9	34	5	0.672	20.4	10.2	100	6	0.632

*#Attribute, the number of attributes; P1st, the largest PPLS*.

**Figure 1:**
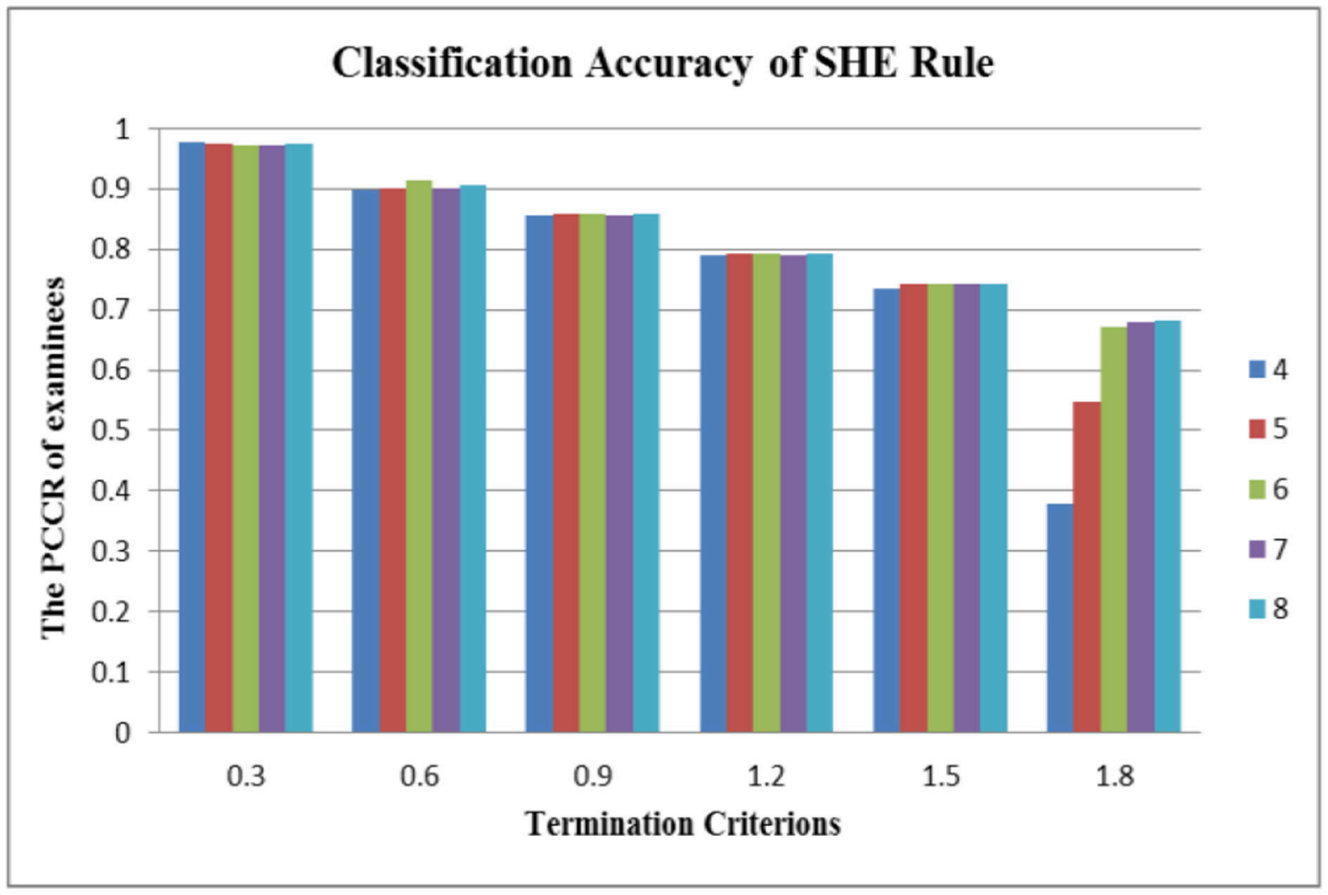
Stability of the SHE rule across different numbers of attributes in the DINA model.

**Figure 2:**
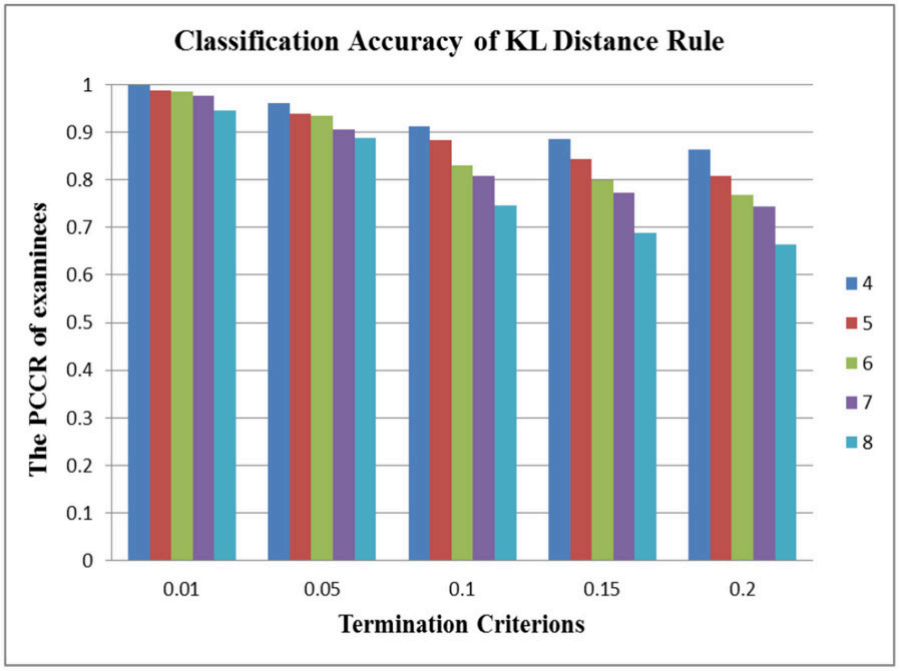
Stability of the KL-distance rule across different numbers of attributes in the DINA model.

In terms of the performance of the absolute and relative approaches across different numbers of attributes, the methods from the absolute approach maintained better stability than those from the relative approach across different numbers of attributes. Table 6 showed that the classification accuracy for different numbers of attributes was approximately the same for both models except when the number of attributes was small (namely, four or five attributes). Figure 1 is the visual representation of this result for the DINA model. Tables 4, 5 indicate that the Tatsuoka rule and the two-criterion rule presented a very similar trend in terms of stability of the classification accuracy across different numbers of attributes. In contrast, those in the relative approach were severely influenced by the number of attributes. Tables 7, 8 indicate that each termination criterion from either the SHE-difference rule or the KL-distance rule produced differential classification accuracy for different numbers of attributes under both models. For example, as shown in Figure 2, under the DINA model, the most conservative termination criterion, 0.01, from the KL-distance rule produced 5% difference in PCCR for four to eight attributes (from 0.998 to 0.945) while the most liberal termination criterion, 0.20, yielded even a more prominent gap of 20% for four to eight attributes (from 0.864 to 0.664). Similar results can be readily identified in the fusion model.

**Table 5:** Classification accuracy for attribute profile and test length using the two-criterion rule.

**#Attribute**	**P1st**	**P2nd**	**DINA**					**FM**				
			**M**	**SD**	**Max**	**Min**	**PCCR**	**M**	**SD**	**Max**	**Min**	**PCCR**
4	0.9	0.1	7.1	2.1	20	3	0.941	12.7	5.2	42	6	0.926
	0.8	0.1	5.5	1.7	18	2	0.867	9.9	4.2	48	5	0.878
	0.7	0.1	4.8	1.3	14	3	0.793	7.7	3.0	24	3	0.777
	0.6	0.1	4.2	0.7	7	2	0.761	6.7	2.7	26	4	0.760
	0.5	0.1	4.2	0.8	8	1	0.796	7.3	2.8	24	4	0.773
5	0.9	0.1	9.3	2.8	27	4	0.934	17.6	7.3	95	7	0.922
	0.8	0.1	7.6	2.2	21	3	0.871	13.8	5.4	51	5	0.842
	0.7	0.1	6.8	1.9	20	3	0.792	11.3	4.6	44	5	0.780
	0.6	0.1	6.0	1.6	17	3	0.765	9.1	3.7	35	4	0.721
	0.5	0.1	6.1	1.8	22	3	0.776	9.4	3.6	27	4	0.732
6	0.9	0.1	12.9	13.5	300	5	0.941	23.1	15.3	300	9	0.913
	0.8	0.1	10.5	7.3	300	4	0.832	18.3	8.0	108	8	0.856
	0.7	0.1	8.5	2.5	28	3	0.787	14.2	8.3	47	6	0.774
	0.6	0.1	7.7	2.3	22	3	0.759	13.6	5.9	96	5	0.735
	0.5	0.1	7.4	2.1	26	4	0.744	11.0	4.2	42	5	0.697
7	0.9	0.1	19.4	15.0	300	6	0.912	31.9	25.0	300	9	0.929
	0.8	0.1	14.2	12.8	300	5	0.850	25.5	17.8	300	8	0.837
	0.7	0.1	10.0	7.5	26	6	0.792	18.5	14.6	50	10	0.753
	0.6	0.1	10.3	3.7	37	5	0.730	17.2	9.5	105	6	0.709
	0.5	0.1	8.0	1.9	28	4	0.675	16.1	7.8	78	6	0.699
8	0.9	0.1	33.8	17.3	300	6	0.917	45.3	40.3	300	10	0.938
	0.8	0.1	25.6	14.9	300	6	0.824	34.0	29.8	300	10	0.853
	0.7	0.1	12.4	3.6	35	6	0.787	24.7	12.8	164	8	0.750
	0.6	0.1	12.4	4.1	38	6	0.706	23.6	12.5	177	7	0.732
	0.5	0.1	10.8	3.5	32	5	0.657	20.4	10.9	114	7	0.656

*P1st, the largest PPLS; P2nd, the second largest PPLS*.

**Table 6:** Classification accuracy for attribute profile and test length using the SHE rule.

**#Attribute**	**ε**	**DINA**					**FM**				
		**M**	**SD**	**Max**	**Min**	**PCCR**	**M**	**SD**	**Max**	**Min**	**PCCR**
4	0.3	8.4	2.7	21	4	0.977	14.8	5.7	63	7	0.974
	0.6	6.4	1.8	16	3	0.899	11.0	4.1	30	5	0.901
	0.9	5.7	1.7	19	3	0.856	9.8	3.4	38	5	0.848
	1.2	4.4	0.9	12	3	0.790	6.8	2.4	19	4	0.782
	1.5	3.9	0.9	10	2	0.734	6.5	2.4	21	3	0.722
	1.8	2.9	0.6	5	2	0.379	4.7	1.6	15	3	0.550
5	0.3	10.6	3.2	28	5	0.976	18.9	7.3	69	8	0.963
	0.6	8.8	2.8	25	4	0.900	15.3	6.4	57	6	0.911
	0.9	7.5	2.4	25	3	0.858	13.8	5.2	44	6	0.842
	1.2	7.1	1.9	21	4	0.793	11.0	4.3	36	5	0.782
	1.5	6.5	1.7	17	3	0.743	9.5	3.6	32	4	0.723
	1.8	4.6	0.8	9	3	0.546	7.8	2.6	26	4	0.644
6	0.3	12.8	7.4	300	6	0.973	17.7	15.7	300	10	0.959
	0.6	11.4	3.3	26	5	0.915	23.1	10.9	136	8	0.908
	0.9	10.5	3.1	25	5	0.859	18.8	7.5	74	6	0.849
	1.2	8.6	2.6	30	4	0.792	15.2	5.7	60	6	0.775
	1.5	8.0	2.6	24	3	0.743	13.3	5.0	43	6	0.727
	1.8	6.8	1.9	20	3	0.671	13.5	5.7	62	5	0.685
7	0.3	15.7	3.6	37	8	0.971	30.0	13.8	300	16	0.961
	0.6	14.1	3.4	38	7	0.901	24.2	11.5	300	13	0.900
	0.9	11.8	2.7	28	7	0.857	21.9	6.2	51	11	0.856
	1.2	10.0	2.0	25	6	0.789	19.7	6.1	61	10	0.774
	1.5	9.0	1.9	25	6	0.743	16.4	4.8	47	9	0.727
	1.8	9.7	3.2	33	4	0.680	16.5	6.6	55	6	0.683
8	0.3	20.0	6.7	58	7	0.974	46.4	24.6	194	11	0.967
	0.6	16.9	5.7	54	6	0.907	40.7	24.3	205	9	0.915
	0.9	15.9	6.1	51	6	0.858	32.4	18.5	231	10	0.860
	1.2	14.7	5.2	50	6	0.792	29.5	17.8	240	8	0.776
	1.5	13.3	5.0	52	5	0.742	25.3	11.8	120	8	0.730
	1.8	12.6	4.8	50	6	0.682	22.6	10.6	122	7	0.690

*ε is the symbol in Equations (4)–(6)*.

**Table 7:** Classification accuracy for attribute profile and test length using the SHE-difference rule.

**#Attribute**	**ε**	**DINA**					**FM**				
		**M**	**SD**	**Max**	**Min**	**PCCR**	**M**	**SD**	**Max**	**Min**	**PCCR**
4	0.01	14.0	3.9	39	7	0.998	24.4	8.2	74	6	0.972
	0.05	10.7	3.1	34	5	0.987	15.8	5.7	39	4	0.896
	0.10	9.8	2.6	26	4	0.967	9.6	4.6	30	3	0.749
	0.15	9.4	2.8	22	2	0.946	6.3	3.3	25	3	0.630
	0.20	7.9	2.2	21	2	0.927	6.5	2.6	17	3	0.672
5	0.01	17.8	5.0	45	5	0.993	28.2	10.7	106	4	0.942
	0.05	12.7	3.6	29	5	0.975	16.3	6.7	49	4	0.794
	0.10	11.6	3.2	29	5	0.926	12.1	4.9	37	2	0.712
	0.15	10.5	3.3	25	3	0.877	6.3	3.8	24	2	0.536
	0.20	8.4	2.1	19	3	0.857	5.6	3.3	19	2	0.503
6	0.01	20.5	6.0	56	7	0.985	31.7	13.3	113	4	0.903
	0.05	14.1	4.7	36	4	0.906	18.1	8.0	58	2	0.746
	0.10	13.4	4.6	32	2	0.864	11.2	5.6	36	2	0.599
	0.15	12.3	3.5	28	4	0.859	6.0	3.0	24	2	0.433
	0.20	10.1	3.5	25	2	0.766	5.3	3.7	27	2	0.353
7	0.01	23.8	5.2	60	9	0.992	40.1	13.4	103	7	0.914
	0.05	17.7	4.2	42	7	0.956	22.1	8.9	54	6	0.744
	0.10	16.1	3.6	38	8	0.912	16.2	6.3	39	5	0.599
	0.15	14.8	3.3	36	7	0.903	8.5	4.9	28	4	0.403
	0.20	12.6	3.3	25	4	0.826	3.9	2.2	21	2	0.211
8	0.01	26.9	9.1	72	5	0.931	37.8	20.2	158	2	0.793
	0.05	16.9	6.5	45	3	0.787	17.2	9.8	76	2	0.539
	0.10	11.1	5.9	36	2	0.542	10.3	5.9	43	2	0.404
	0.15	9.7	4.8	31	3	0.485	5.4	2.8	26	2	0.218
	0.20	8.2	3.9	24	2	0.433	4.1	2.0	21	2	0.229

**Table 8:** Classification accuracy for attribute profile and test length using the KL-distance rule.

**#Attribute**	**ε**	**DINA**					**FM**				
		**M**	**SD**	**Max**	**Min**	**PCCR**	**M**	**SD**	**Max**	**Min**	**PCCR**
4	0.01	10.3	2.8	26	5	0.998	15.6	5.4	50	8	0.978
	0.05	8.7	2.6	28	4	0.962	11.1	4.4	37	5	0.890
	0.10	6.7	2.0	20	4	0.913	8.8	3.2	28	4	0.790
	0.15	6.2	2.0	23	4	0.886	4.5	2.2	15	2	0.528
	0.20	6.1	1.7	17	4	0.864	3.9	1.4	10	2	0.516
5	0.01	13.3	3.9	36	6	0.988	23.1	8.4	65	8	0.966
	0.05	10.5	3.3	30	4	0.939	14.4	5.0	46	6	0.896
	0.10	8.7	2.7	27	4	0.884	10.3	3.6	29	3	0.738
	0.15	7.6	2.2	24	3	0.843	7.2	2.1	17	3	0.642
	0.20	7.5	2.1	19	4	0.809	4.2	1.5	12	2	0.435
6	0.01	15.8	4.5	56	6	0.985	26.5	10.0	92	10	0.953
	0.05	12.5	3.4	34	3	0.934	17.9	6.1	42	7	0.851
	0.10	9.6	2.6	25	3	0.830	11.9	4.1	36	3	0.678
	0.15	9.3	2.4	25	3	0.800	5.6	3.2	19	2	0.401
	0.20	8.5	2.0	24	2	0.768	5.6	1.8	14	2	0.386
7	0.01	17.2	3.9	36	10	0.976	29.7	8.2	86	16	0.931
	0.05	14.0	3.1	41	8	0.905	18.4	5.3	60	6	0.740
	0.10	11.3	2.7	29	7	0.809	9.4	3.4	27	5	0.466
	0.15	10.0	2.3	25	6	0.772	7.0	2.3	19	4	0.408
	0.20	9.8	2.2	23	6	0.743	3.4	2.1	14	2	0.170
8	0.01	21.4	6.1	51	10	0.945	39.5	13.0	92	13	0.912
	0.05	16.0	4.5	45	8	0.887	22.0	6.6	55	5	0.710
	0.10	12.6	3.6	33	4	0.747	14.4	4.1	40	2	0.574
	0.15	12.1	3.2	32	3	0.688	6.8	3.3	22	2	0.289
	0.20	11.1	2.5	25	2	0.664	3.8	2.4	13	2	0.152

**Figure 3:**
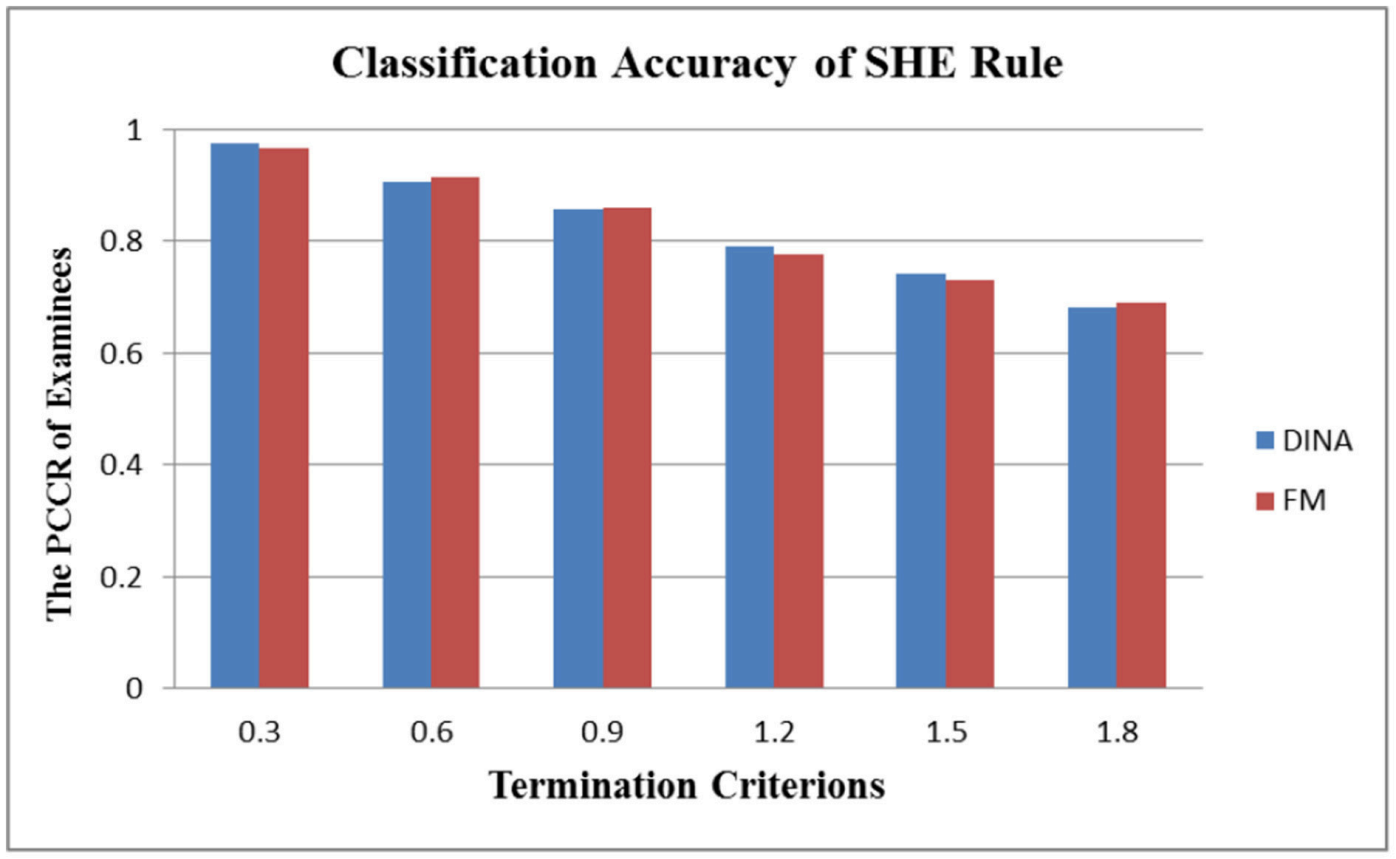
Stability of the SHE rule across different models for eight attributes.

**Figure 4:**
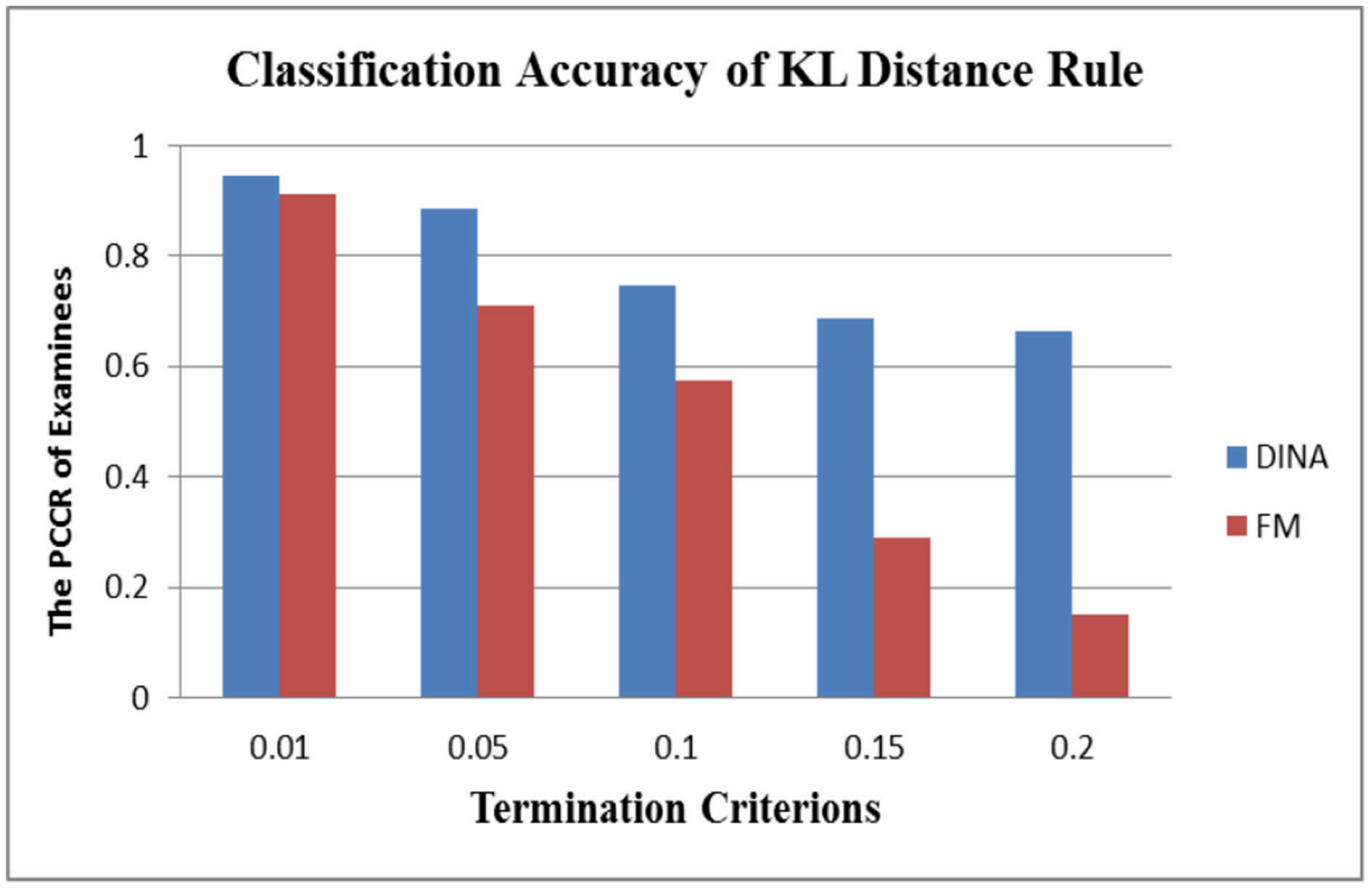
Stability of the KL-distance rule across different models for eight attributes.

In the aspect of cross-model stability, the differential performance between the absolute and relative approach was even more striking. Take the SHE rule and the KL-distance rule as an example. Figures 3, 4 show the classification accuracy for the SHE rule and KL-distance rule under the DINA and fusion models for eight attributes. The SHE rule produced similar classification accuracy for both models under all the different termination criteria while the KL-distance rule yielded drastically different classification accuracy for the two models.

In summary, the absolute approach, two previous methods, and the SHE rule did a much better job than the relative approach in terms of stability across differing numbers of attributes and different CDMs.

Simulation study 1 also provided some preliminary result for the partial-vs.-full information comparison. Within the absolute approach, the full information approach (the SHE rule) was slightly more consistent with respect to the classification accuracy than the partial information approaches (the Tatsuoka rule and the two-criterion rule). More interestingly, there are reversed classification accuracies for both the Tatsuoka rule and the two-criterion rule. For example, Table 4 shows that for the DINA model with four attributes, the classification rate for the termination criterion 0.6 is 0.739, which is smaller than 0.752, the one for the criterion 0.5. The two-criterion rule suffered from this problem for both models with four and five attributes as shown in Table 5.

To further reveal the differential performance between the partial-vs.-full information approaches, Study 2 attempted to explore this issue under a more realistic application setting with a larger number of attributes.

### Study 2: Full vs. Partial Information

#### Design

Study 2 aimed to investigate the performance of the absolute full information approach (the SHE rule) and the absolute partial information approach (the two-criterion rule) for a large number of attributes. As shown from the results of the study 1, classification accuracies were certainly high when termination criteria were set at stringent levels; we do not expect too much difference among these methods which echoes the first practical recommendation for *P*_1st_ and *P*_2nd_ from Hsu et al. (2013). In order to better investigate the performance of full and partial information approaches with respect to stability, more liberal termination criteria should be adopted. The termination criteria for the SHE rule were changed to 1.6, 1.8, and 2.0. The three termination criteria for the two-criterion rule were 0.6, 0.7, and 0.8. In addition, from a practical perspective, the number of attributes can be as many as 14 (McGlohen and Chang, 2008; Jang, 2009; Roman, 2009), and the classification accuracy of the attribute profile is not necessarily as high as 0.8 or even 0.9 since formative assessment is usually low-stakes. Hence, the number of attributes was set to be either 8, 10, or 12. Thus, the performance of the termination rules under these conditions carries a practical implication. Since there are more attributes, a larger item pool is needed for the simulation. The item pool used in study 2 was generated in the same way as in study 1 except that it consisted of 1,000 items instead of 300 items. Due to the large number of attributes, it might take a lot of items for some examinees to finish the test, so the maximum number of items an examinee can take in a CD-CAT test was set to 100, which was 10% of the total number of items.

The basic setup for study 2 was similar to that for study 1. There were three factors in this simulation study: CDMs, number of attributes, and termination rules. The major dependent variables were the same as in study 1. The ratio of examinees who reached the maximum test length as a confounding variable was also reported.

#### Results

Tables 9, 10 summarize the results for the simulation. The 12-attribute condition showed that the proportions of examinees reaching the maximum test length in the two-criterion rule were higher than those in the SHE rule under both models in the corresponding conditions. Beyond that, most results indicated that the proportions of examinees attaining the maximum test length were small under a variety of conditions in the study, so this confounding variable was well-controlled. The effect of the proportion of examinees using the maximum length stopping rule will be discussed in detail in the Discussion section. The eight-attribute condition can be considered as a partial replication study of study 1 since the only difference is the bank size, which increased from 300 to 1,000. The results for this condition were very similar to those from study 1 and thus the possible confounding bank effect was also eliminated from study 2.

**Table 9:** Summary statistics for the SHE rule.

**#Attribute**	**ε**	**DINA**						**FM**					
		**M**	**SD**	**Max**	**Min**	**%(ML)**	**PCCR(P)**	**M**	**SD**	**Max**	**Min**	**%(ML)**	**PCCR(P)**
8	1.6	12.4	3.2	32	5	0	0.732	18.6	6.2	53	7	0	0.737
	1.8	11.1	2.8	32	4	0	0.682	17.0	6.1	59	7	0	0.708
	2.0	10.2	3.1	30	4	0	0.640	16.4	5.9	52	6	0	0.654
10	1.6	16.9	7.6	100	7	0.3	0.733	28.2	11.2	100	10	0.05	0.739
	1.8	15.3	6.1	100	6	0.15	0.692	27.7	11.5	95	9	0	0.710
	2.0	15.3	6.3	100	6	0.15	0.642	26.5	10.0	88	8	0	0.652
12	1.6	27.2	20.2	100	8	6	0.733	42.3	18.1	100	13	2	0.733
	1.8	26.2	20.9	100	8	6.4	0.691	37.3	16.7	100	12	1	0.709
	2.0	23.6	17.6	100	9	4	0.645	39.6	17.6	100	11	1	0.653

*%(ML) = %(Max Length), the ratio of examinees attaining the maximum test length; PCCR(P), the pattern correct classification rate for examinees who finished the CD-CAT using termination rules*.

**Table 10:** Summary statistics for the two-criterion rule.

**#Attribute**	***P*_**1st**_**	***P*_**2nd**_**	**DINA**						**FM**					
			**M**	**SD**	**Max**	**Min**	**%(ML)**	**PCCR(P)**	**M**	**SD**	**Max**	**Min**	**%(ML)**	**PCCR(P)**
8	0.8	0.1	13.0	3.8	34	6	0	0.829	23.5	8.0	67	9	0	0.843
	0.7	0.1	11.9	3.5	33	6	0	0.801	20.3	7.1	64	7	0	0.787
	0.6	0.1	10.3	2.6	25	4	0	0.747	17.6	6.3	54	7	0	0.709
10	0.8	0.1	21.3	12.7	100	8	2	0.849	33.7	13.3	100	12	0.2	0.843
	0.7	0.1	18.6	12.4	100	8	1.8	0.768	30.1	12.8	100	10	0.1	0.779
	0.6	0.1	17.8	13.4	100	7	2.2	0.706	27.3	11.9	91	10	0	0.683
12	0.8	0.1	31.0	23.6	100	11	9.7	0.858	48.3	20.6	100	13	4.9	0.867
	0.7	0.1	30.7	25.5	100	11	11.2	0.750	43.5	19.1	100	12	3.2	0.784
	0.6	0.1	26.3	23.1	100	9	8.4	0.677	40.3	18.2	100	11	1.8	0.738

This simulation produced similar results for the two rules under the large number of attributes to study 1. The SHE rule demonstrated strong stability across both the number of attributes and the CDMs while the two-criterion rule had some irregularity for some conditions. The classification accuracy for the three numbers of attributes was almost equal to 0.73, 0.70, and 0.65, respectively, for three termination criteria (1.6, 1.8, and 2.0). However, some termination criteria from the two-criterion rule yielded different classification accuracies for different numbers of attributes. For example, for the termination criterion *P*_1st_ = 0.6, the classification accuracy under the DINA model was 0.747, 0.706, and 0.677, respectively, for three different numbers of attributes (8, 10, and 12). Similar results can be easily identified for the fusion model.

In terms of cross-model constancy, the SHE rule also presented strong stability of classification accuracy. The two-criterion rule improved, but there were also inconsistencies of classification accuracy between the DINA model and the fusion model. The biggest difference was equal to 0.061 (= 0.738–0.677), which appeared on the condition of *P*_1st_ = 0.6 and 12 attributes.

## Discussion

Cognitive diagnostic assessment (CDA) informs an examiner about the attribute mastery pattern of every student so that designing effective remedial interventions in formative instruction can be administered (Leighton and Gierl, 2007a; Cui et al., 2012). CD-CAT as the computerized adaptive version of the CDA needs a flexible termination rule that can stop the test at an appropriate level to achieve that goal. This study provided a theoretical derivation of information-based termination rules proposed by Cheng (2008) and demonstrated the instability issue with previous methods from the information theory perspective. Two multi-factor simulation studies were conducted to evaluate the new three termination rules.

Some important observations can be made. The first point worth noting is that not all the full information methods outperform the previous methods, and the absolute full information method, the SHE rule, is the best with regard to the cross-attribute and cross-model stability. From the two simulation studies, we identified the termination criteria for the termination rule ranging from 0.3 to 1.8, which could produce a smooth decreasing trend of the estimate accuracy from about 0.97–0.6. The classification accuracy was not affected by the number of attributes (if it is more than five) or by the models. This implies that the SHE rule is a very flexible and effective method to stop the variable-length CD-CAT.

Then, there are some common problems shared by the Tatsuoka rule and the two-criterion rule. First, they are affected by the number of attributes, although their between-model performances are decent. Some careful consideration must be given with regard to the number of attributes for the item pool. Second, if some liberal criteria are used, such as *P*_1st_ is 0.6, 0.7, or 0.8 for large numbers of attributes, the problem of instability across different numbers of attributes is exacerbated. This reflects the inherent problem with the partial information rules. In CDA, the number of attribute patterns increases exponentially with the number of attributes. For a large number of attributes, the partial information rules do not have an effective control and thus there is a wide range of classification accuracy for differing numbers of attributes, although they, as members of the absolute approach, can guarantee a lower bound of the classification accuracy as the SHE rule does.

Lastly, the use of the maximum test length rule, in combination with the variable-length termination rule, and the proportion of examinees using this rule are important in the variable-length CD-CAT application. As noted above, the number of attribute profiles increases exponentially with that of attributes. When the number of attributes is large, the number of attribute profiles is so huge that it will take a lot of items—in some instances, even the entire item pool—for some examinees to satisfy the requirement prescribed by the termination rule. Thus, it is necessary to set the maximum test length even if a variable-length termination rule is adopted, and this treatment is often imposed in real CAT programs. It is also necessary to monitor the proportion of examinees hitting the maximum test length. If that proportion is high, then there might be some problems that merit further investigation, such as the criterion for the variable-length termination rule being too conservative, or there not being enough high-quality items in the pool. One possible solution to this issue is to make use of the attribute hierarchical structure (Leighton and Gierl, 2007b) to cut down the number of possible attribute profiles and then construct an informative prior for the distribution of the attribute profile.

Several issues require further investigation. In real testing situations, different CDMs, different item selection algorithms, item exposure control methods, content and attribute balancing, and item pool quality are all possible elements that could affect the performance of all the rules; more simulation studies are needed to investigate these situations. In the current study, we only used the DINA and fusion model as examples and the result with regard to the cross-model stability should be interpreted with caution. We carefully chose the two models that are the two ends of the spectrum of the existing CDMs and a similar conclusion regarding the SHE rule is expected for other models, but further study in this aspect is still warranted. Although two simulation studies were conducted, some real-life data studies are also necessary to investigate the performance of these termination rules in real situations. In addition, as one anonymous reviewer pointed out, the simulated examinees would answer an average of only 26% of the items correctly using the procedure described in the first simulation study based on the DINA model with eight attributes. There could be some reasons for this, such as the quality of item pool, the Q-matrix of test, distribution of attribute profiles in the population, and CDMs. An interesting study in the future is to investigate how the generation procedures of examinees' attribute profiles affect the classification accuracy and responses.

## Author Contributions

LG proposed the idea of this article and wrote all the simulation study code. CZ is the corresponding author who mainly organized and wrote the article.

### Conflict of Interest Statement

The authors declare that the research was conducted in the absence of any commercial or financial relationships that could be construed as a potential conflict of interest.
